# Bacterial Vaccine Antigen Discovery in the Reverse Vaccinology 2.0 Era: Progress and Challenges

**DOI:** 10.3389/fimmu.2018.02315

**Published:** 2018-10-08

**Authors:** Fadil A. Bidmos, Sara Siris, Camilla A. Gladstone, Paul R. Langford

**Affiliations:** Department of Medicine, Imperial College London, London, United Kingdom

**Keywords:** reverse vaccinology 2.0, human monoclonal antibodies, bacterial pathogens, vaccine candidate antigens, immunotherapy

## Abstract

The ongoing, and very serious, threat from antimicrobial resistance necessitates the development and use of preventative measures, predominantly vaccination. Polysaccharide-based vaccines have provided a degree of success in limiting morbidity from disseminated bacterial infections, including those caused by the major human obligate pathogens, *Neisseria meningitidis*, and *Streptococcus pneumoniae*. Limitations of these polysaccharide vaccines, such as partial coverage and induced escape leading to persistence of disease, provide a compelling argument for the development of protein vaccines. In this review, we briefly chronicle approaches that have yielded licensed vaccines before highlighting reverse vaccinology 2.0 and its potential application in the discovery of novel bacterial protein vaccine candidates. Technical challenges and research gaps are also discussed.

## Background: vaccine discovery in the pre-whole genome sequencing (WGS) ERA

Precedent to advancements in genomics, vaccines were developed based on Pasteur's *rules of vaccinology*. A 230-fold serial passage of a bovine bacillus in bile medium produced the live attenuated Bacillus Calmette-Guerin (BCG) vaccine against *Mycobacterium tuberculosis* (MTB) (1). Then, a trivalent blend of poliovirus inactivated in < 0.5% formalin was used by Salk et al. as a safe and effective vaccine against poliovirus (2). The inability to culture some pathogens *in vitro* (owing to safety or lack of suitable culture conditions), extensive antigenic variability, and molecular mimicry limit the broad applicability of traditional culture-based techniques in the development of vaccines targeting other economically-important pathogens such as *Mycobacterium leprae* and *Neisseria meningitidis*.

The inadequacies of culture-based techniques caused a shift in focus to the use of subunit components as vaccine candidates. Identification of these subunit vaccine candidates was largely hypothesis-driven, targeted cellular components and were often well-known virulence factors: for example, the pertussis toxin and fimbriae in the acellular pertussis vaccine (3); and the meningococcal porin, PorA, in the epidemic-specific detergent-extracted outer membrane vesicle (OMV) vaccines of Chile, Norway, and New Zealand (4–6). With complex biosynthetic methods, bacterial capsular polysaccharides served as prime components of effective vaccines, used singly, for example, in the 23-valent pneumococcal polysaccharide vaccine, PPSV23 (7). In addition, these capsular antigens have been conjugated to carrier proteins in a dose-sensitive manner for enhanced immunogenicity, as found in the *Haemophilus influenzae* type b (Hib) (8), pneumococcal (9), and meningococcal (10) conjugate vaccines. The limited capacity of these hypothesis-driven studies to focus on only a handful of candidates at a time was costly in time, labor and financial terms. This is especially because of the large pool from which prospective candidates for individual bacterial pathogens are screened, coupled with the low likelihood of targets satisfying key vaccine candidacy criteria (abundantly-expressed, surface-exposed, functionally-immunogenic, and highly-conserved). Thus, alternative high throughput methods were sought to accelerate the pre-clinical vaccine development phase, especially in situations requiring rapid curtailment of disease transmission.

## Whole genomic and proteomic approaches

### Reverse vaccinology (RV)

The publication of the first complete bacterial genome sequence in 1995 [for *H. influenzae* (11)] heralded a revolution in approaches to vaccine development. By using genomic data and preset bioinformatic screens, putative surface-associated antigens of a pathogen were identified. The subsequent recombinant expression of these genes and immunization of animals with recombinant proteins, for the determination of active and passive levels of protection, provided data that substantiated or annulled the vaccine candidacy of selected antigens (12, 13). This “classical” RV approach led to the development of the multicomponent meningococcal serogroup B vaccine (4CMenB) (14). While 4CMenB has potential for cross-serogroup protection (15), it has been argued that pan-genomic *in silico* analysis is more appropriate because of the high degree of intraspecific diversity exhibited by many bacterial pathogens (16). Using this pan-genomic approach, Maione et al. (17) identified four protective antigens from the analysis of an octa-genomic panel derived from the most prevalent disease-causing *Streptococcus agalactiae* strains. The main attraction of RV lies in its applicability to any pathogen with WGS data and to which antibody-mediated immunity for protection against disease is crucial. Its use in the discovery of candidate antigens comprising vaccines targeting other bacterial pathogens, including the multidrug-resistant *Acinetobacter baumanii*, has been demonstrated (18–20). However, important non-classical surface-associated proteins may be missed due to the parameters of the bioinformatic screen(s).

Related to RV is the use of transcriptomics to identify novel vaccine antigens. For example, the comparative analysis of the meningococcal transcriptome in *ex vivo* human whole blood and *in vitro* nasopharyngeal colonization models revealed three antigens that were differentially regulated between invasive disease and asymptomatic colonization, and were thus subjects for further vaccine candidacy studies (21) However, this transcriptomics-based approach has not been widely employed.

### Surfome and secretome analysis

Whole proteomic approaches, involving enzymatic processing of whole cells or extracellular exudates followed by liquid-chromatography mass spectrometry (LC-MS) or peptide fragment fingerprinting, also allow for high-throughput screening of the antigenic repertoire of a pathogen (22). The power of these proteomic methods in identifying rare protective antigens missed by the *in silico* screens of RV makes them appealing [as exemplified by the case of the cell wall-anchored antigen, SAN_1485, of *S. agalactiae* (23)]. Converse to RV, proteolytic digestion is more suited toward Gram-positive bacteria, since Gram-negative bacteria are more susceptible to proteolysis-induced cell lysis.

## Reverse vaccinology 2.0

The majority of currently-available bacterial vaccines provide protection by inducing pathogen-specific antibodies. Therefore, harnessing the antibody component of a potent human humoral response to disseminated infection is valuable for the identification of novel protective antigens. This approach, termed reverse vaccinology 2.0 (RV 2.0) (24, 25), relies on the isolation and recombinant expression of the variable regions of heavy (VH) and light (VL = κ or λ) chain genes of immunoglobulin (focus has centerd on IgG) using a variety of molecular tools. Enriched by the development of high-throughput technologies, the screening of large numbers of antibody-secreting cells (ASCs) is also advancing knowledge of host-pathogen interactive biology and auto-immunity (26, 27).

### Monoclonal antibody (mAb) generation from ASCs

The first, and perhaps most crucial, phase of RV 2.0 is the cloning of human monoclonal antibodies (mAbs) from ASCs. Previously, immortalization of these ASCs via myeloma fusions or Epstein Barr virus (EBV) transformation were valuable to mAb production (28, 29). Because these were culture-based methods, the survival of all B-cells was not guaranteed and the omission of ASCs expressing antibodies cognate to crucial antigens was probable. Other techniques such as phage-display technology (30) and proteomic mining (31, 32) circumvent the unique issues affecting ASC immortalization techniques by focusing on recombinant antibody expression. However, the small proportion of antigen-specific antibodies (estimated at 10–15%) that are produced (33) because of the random pairing of VH and VL sequences make phage display and proteomic mining imprecise.

A more favored approach to mAb cloning is the single-cell sorting of ASCs into multi-well plates using flow cytometry, followed by the cloning of mAbs from each well (34, 35). To clone a high proportion of antigen-specific antibodies, this approach, termed expression cloning, requires blood sampling during the peak immune response and is thus more suited to short-lived plasmablasts (CD3^−^, CD14^−^, CD19^+^, CD20^−^, CD56^−^, CD27^high^, and CD38^high^), since higher circulating numbers of these are indicative of very recent history of infection (36). Notwithstanding, several studies have demonstrated its applicability to memory B-cells (37, 38). Further *in vitro* selection of antigen-specific plasmablasts or memory B-cells using eGFP-bound viral-like particles (39), labeled-antigen probes (40, 41) or *in vivo* antigen-specific plasmablast enrichment in irradiated SCID/beige chimera mice (42) enhance the pathogen-specific mAb output of the approach. Converse to phage display and proteomic mining technologies, expression cloning yields mAbs with natural, host-like VH+VL pairings. Further refinements to this elegant method include: substituting restriction endonuclease cloning with Gibson assembly to enhance cloning precision (43); assembly of both VH and VL fragments into a single expression vector (44); and succeeding cell sorting with paired-chain antibody repertoire sequencing, thereby encompassing all V gene families, including unique clones expressed at low frequencies (33, 45, 46).

### Assessment of recombinant mAb function

Subsequent to cloning, the clinical relevance of mAbs is assessed in *in vivo* investigations of passive immunity (47) or *in vitro* functional assays: for example, the well-established viral neutralization (48) and serum bactericidal assays (49), some of which have provided data employed in vaccine licensure (50). The cognate antigens targeted by functional mAbs can subsequently be determined using protein array screens or classical immunoproteomic approaches.

### Application of RV 2.0 to viral vaccine development

**Figure 1:**
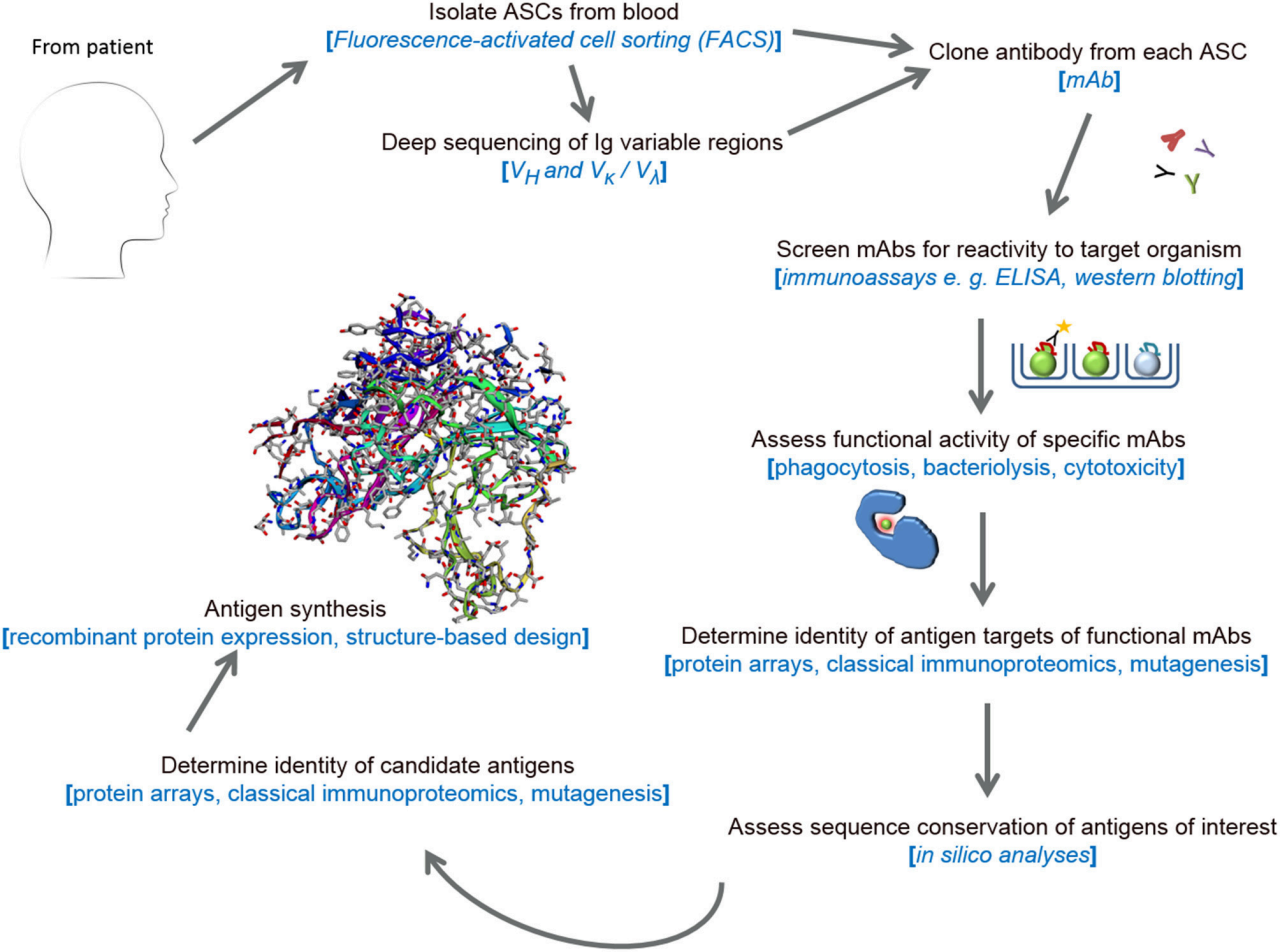
Schematic representation of RV 2.0.

The power of RV 2.0 (see Figure 1) in the identification of viral vaccine candidates has been demonstrated in several studies focussing on human cytomegalovirus (HCMV), respiratory syncytial virus (RSV), HIV, influenza and dengue viruses (25, 51). Some of these candidate antigens, discovered using RV 2.0, include a novel pentameric glycoprotein complex, the gHgLpUL128L pentamer, which induces high neutralizing titres against HCMV in mice (52) and the F protein of RSV stabilized to the prefusion conformation (53, 54). Accruing data from phase 1b/2a clinical trials show that a mAb (MEDI8897) reactive with prefusion F epitopes is effective when used prophylactically in preterm infants (55). Like MEDI8897, mAb MHAA4549A, cloned from a healthy vaccinee and which targets and neutralizes all known influenza A strains (56), demonstrated significant antiviral activity in a phase 2 human influenza A virus challenge (57). Thus, these studies have signified the use of RV 2.0 in producing broadly-neutralizing mAbs for post-infection prophylaxis in addition to identifying functionally-immunogenic vaccine candidates.

## Potential application to antibacterial vaccine discovery

Judging by the progress made with the development of novel and effective viral immunotherapies, RV 2.0 is showing promise and is equally applicable to bacterial vaccinology. RV 2.0 was employed by Lu et al. (58) to identify functional anti-*Staphylococcus aureus* mAbs induced during bacteraemia. A total of ten mAbs were produced, four of which enhanced opsonophagocytosis of Wood46, a *S. aureus* reference strain. While three of the four functional mAbs targeted *S. aureus* antigens with known identities, the fourth mAb reacted with a novel antigen. Recently, Zimmermann et al. (59) also demonstrated that functional anti-MTB surface antigen antibodies can be cloned from patient-derived plasmablasts of reactivated memory B-cell origins, providing further evidence for a role for antibodies in the modulation of potent immune responses toward MTB. Taken together with other studies investigating the importance of antibody-mediated neutralization of intracellular pathogens (60), a role for the vaccine-induced generation of antibodies against pathogens such as MTB and *Chlamydia trachomatis*, using antigens derived with RV 2.0 is, thus, evidenced. Similarly, Bidmos et al. (61) and Blum et al. (45) cloned functional antibodies from sufferers of meningococcal and Lyme disease, respectively; thus, underscoring the utility of the approach for identifying novel targets in different classes of bacteria. Continued use of RV 2.0 in bacterial vaccine discovery is, therefore, encouraged following the surmounting of technical challenges and filling of research gaps. In the following sections of this mini-review, emphasis will be placed on human mAb cloning and serological correlates of protection, since other related technical aspects of RV 2.0 such as recombinant protein expression, high-throughput sequencing of bacterial genomes and antibody repertoires, antigen identity determination and structure-based antigen design have been reviewed elsewhere (62–66).

### Pathogen-specific mAb output

To identify novel antigens using the expression cloning method with precision, plasmablasts from patients convalescing from bacterial disease are required. Fundamental to the application of the expression cloning approach, therefore, is the determination of the magnitude and peak duration of the plasmablast response in these patients. The information on the duration of peak plasmablast circulation instructs optimum sampling time, which in turn impacts on the precision of pathogen-specific mAb generation. Studies assessing this magnitude of circulating plasmablast following bacterial infection have reported similar durations of peak response to those reported for primary or secondary viral infections [6–7 days for primary infections and ~10 days post-infection for secondary infections; reviewed in (36)]. Recently, Band et al. (67) reported a significant induction of differentiating (Ki-67^+^) plasmablasts in patients of nosocomial bacterial infections compared to healthy controls. This induction peaked between days 8 and 16 post-culture positivity in *A. baumanii*-infected patients reaching levels as high as 21% of the total lymphocyte population. Perhaps unsurprisingly, it was also observed that this induction was markedly different in individuals, reflecting differences in immunocompetence, as peak plasmablast levels ranged from: 1 to 21% among *A. baumanii*-infected patients; and 5–40% in *Escherichia coli*-infected patients. Consistent with the findings of Band et al. (67), a plasmablast response presented to: MTB infection in 38% of a patient cohort with levels ranging from 1 to 4% in those with strong serum IgG responses (59); *S. aureus* bacteraemia with mean levels of ~3.2% (1–7% range) (58); and up to 4% of circulating CD19+ cells in untreated sufferers of Lyme borreliosis (45).

The implications of these data for the precision of the pathogen-specific mAb output are considerable. Firstly, there is a paucity of information in published literature on how many plasmablasts are pathogen-specific (bacterial) following inductions in patients, owing to the unavailability of suitable molecular probes that will enhance the Fluorescent Activated Cell Sorting (FACS) gating strategy. In the absence of such data, strategies such as the Ig-capture based technique described by Pinder et al. (40) could be employed to enrich for specific plasmablasts. It is more likely that complex antigens (whole bacterial cells, OMV, or outer membrane preparations) would be more beneficial in these strategies, when adapted, compared to single-antigen probes in order to obtain a plasmablast population targeting a wider antigen pool. It is noteworthy that in cases where patients are subjected to immediate antibiotic therapy on hospital admission because of rapid progression of disease (e.g., septicaemia and meningitis), clinical isolates may be unobtainable, making the design of plasmablast enrichment probes difficult (also, the reason behind the unsuitability of memory B-cells in the absence of enrichment strategies). While clinical isolates from other disease sufferers could be utilized, they are non-ideal because rare mAb epitopes specific for the infecting strain will be missed. Secondly, considering differences in the magnitude of the plasmablast response and for logistic reasons (for example, restrictions on blood sample volume in pediatric cases), pooling of patient samples may be required for the generation of a highly-diverse plasmablast pool, targeting several antigens, (and their variants) of the same pathogen. This is especially necessary for pathogens in which certain antigens are immunodominant such as PorA of *N. meningitidis*, which may mask immunity to rare but equally protective antigens.

If a total plasmablast sort approach is warranted (i.e., inclusive of non-pathogen specific plasmablasts), an attractive option is the rational selection of over-represented VH+VL combinations for mAb cloning based on the assumption that overrepresentation of V families, specifically among plasmablasts, is an indicator of preferential usage in response to a pathogen. Adequate depth of sequencing is, however, required in order to avoid non-inclusion of clonal V families expressed at lower frequencies (58). *In silico* analysis should also include antibodies with similar complementarity-determining region H3 loops [key to antibody conformation and affinity (68)] in addition to the exploitation of de-noising algorithms, which would minimize the presence of errors introduced by sequencing (69).

### *In vitro* serological correlates of protection

Assessment of pathogen-specific mAb function is performed via standardized assays. Given the differences in biology of bacterial pathogens, these assays are specifically tailored to reflect mode of clearance of the pathogen from systemic circulation. Antibody-driven, complement-dependent bactericidal activity is measured in the standardized serum bactericidal assays designed for the meningococcus (49) while phagocytosis by neutrophils and macrophages enhanced by opsonic antibodies is assessed in the opsonophagocytic assays used in pneumococcal vaccine development (70). Similar assays have been employed in the assessment of functional immunity against *Campylobacter jejuni*, Group B *Streptococcus*, typhoidal and non-typhoidal *Salmonella* and *Neisseria gonorrhoeae* (71–75). While standardization of some of these pathogen-specific assays is pending, *de novo* design of *in vitro* correlates assessing functional activity of antibodies is not as straightforward for other pathogens, such as *Bordetella pertussis* (76). For facultative intracellular pathogens such as *Francisella tularensis* and MTB, for example, current correlate strategies in development are not suitable for assessments of mAb function as they involve peripheral blood lymphocytes only (77, 78). An added benefit of *in vitro* assessments of cloned mAb function, as a component of RV 2.0, is the needlessness of or significant reduction in usage of experimental animals. Efforts to develop and standardize *in vitro* correlates to assess mAb function are, hereby, merited.

Beyond bactericidal or opsonic functions, mAbs exhibit a variety of functions, including the modulation of cellular immune responses [extensively reviewed in Cooper (79), Amanna and Slifka (80)], which require assessment. These functions also include toxin neutralization (useful in pertussis and diphtheritic infections, for example) (81, 82) and increase in cellular cytotoxicity affecting intracellular pathogens such as *C. trachomatis* (83). Hence, non-bactericidal or non-opsonic mAbs, if exhibitive of these other functions, can still be utilized in other immunotherapeutic avenues.

## Conclusion

With the increase in multidrug resistance among bacterial pathogens, the development of further effective preventive measures will be of significant benefit to public health. RV 2.0, a conceptually-advanced approach with the advantages of employing the natural host response (patient VH-VL combinations), relative speed, and reduction in animal use, has the potential to be a powerful tool in bacterial vaccine development. However, use of RV 2.0 is dependent on optimization of the technical aspects, and there are excellent prospects that this is achievable.

## Author contributions

FB and PL conceptualization. FB, SS, and CG writing—original draft. FB and PL writing—reviewing and editing.

### Conflict of interest statement

The authors declare that the research was conducted in the absence of any commercial or financial relationships that could be construed as a potential conflict of interest.
